# Enhanced Reprogramming Efficiency and Kinetics of Induced Pluripotent Stem Cells Derived from Human Duchenne Muscular Dystrophy

**DOI:** 10.1371/currents.md.a77c2f0516a8cb4809ffad5963342905

**Published:** 2015-09-03

**Authors:** Pooja Teotia, Sujata Mohanty, Madhulika Kabra, Sheffali Gulati, Balram Airan

**Affiliations:** Stem Cell Facility, All India Institute of Medical Sciences, N. Delhi, India; Stem Cell Facility, All India Institute of Medical Sciences, N. Delhi, India; Genetics Unit, Department of Pediatrics, All India Institute of Medical Sciences, N. Delhi, India; Child Neurology Division, Department of Pediatrics, All India Institute of Medical Sciences, N. Delhi, India; Department of Cardio Thoracic Vascular Surgery, All India Institute of Medical Sciences, N. Delhi, India

## Abstract

The generation of disease-specific induced pluripotent stem cells (iPSCs) holds a great promise for understanding disease mechanisms and for drug screening. Recently, patient-derived iPSCs, containing identical genetic anomalies of the patient, have offered a breakthrough approach to studying Duchenne muscular dystrophy (DMD), a fatal disease caused by the mutation in the dystrophin gene. However, development of scalable and high fidelity DMD-iPSCs is hampered by low reprogramming efficiency, the addition of expensive growth factors and slow kinetics of disease-specific fibroblasts. Here, we show an efficient generation of DMD-iPSCs on bFGF secreting human foreskin fibroblast feeders (I-HFF) by employing single polycistronic lentiviral vector for delivering of transcription factors to DMD patient-specific fibroblast cells. Using this method, DMD-iPSCs generated on I-HFF feeders displayed pluripotent characteristics and disease genotype with improved reprogramming efficiency and kinetics over to mouse feeders. Moreover, we were able to maintain disease-specific iPSCs without additional supplementation of bFGF on I-HFF feeders. Our findings offer improvements in the generation of DMD-iPSCs and will facilitate in understanding of pathological mechanisms and screening of safer drugs for clinical intervention. Key Words: Duchenne Muscular Dystrophy, Reprogramming, Induced pluripotent Stem Cells, Immortalized Human Feeder, Basic Fibroblast Growth Factor, Stem Cell Cassette

## Introduction

Disease-specific induced pluripotent stem cells (iPSCs) from patients with incurable diseases have come up with promising approach for research and clinical use in disease modeling including Duchenne muscular dystrophy (DMD), a disease caused by a mutation in the dystrophin gene 1 ^,^ 2 ^,^ 3. Innovations in iPSC technology have enabled us to generate an unlimited autologous number of cells for disease modeling with identical genetic anomalies with an endogenous regulatory system of disease 4 ^,^ 5. However, cellular changes during the reprogramming process, culture-induced differentiation, and differences in the genetic background limit their use in disease-specific phenotypes 6. Additionally, generation of disease-specific iPSCs is also surrounded by various impeding factors like inefficiency, slow kinetics, variability in protocols, and the high cost of the cell culture maintenance 7 ^,^ 8. Many approaches have been developed to overcome these limitations. The initial methods for reprogramming of DMD-iPSCs were based on the monocistronic approach which may lead to leaky expression of reprogramming factors and low reprogramming efficiency 9 ^,^ 1 ^,^ 10 ^,^ 11. Contrary to this, polycistronic lentiviral delivery offers the improvement over the monocistronic approach by synthesis of all four reprogramming factors from one mRNA to generate DMD-iPSCs 12 ^,^ 13. In addition to the transcription factors, suitable techniques of factor delivery are crucial in order to use of iPSCs for clinical applications 14.

Various viral delivery methods such as integrating viral methods i.e. retroviral monocistronic 15, lentiviral polycistronic with or without cre-lox mediated transgene excision 16 ^,^ 17; non-integrating viral methods i.e. adenovirus 18, Sendai virus 19; and non-viral methods i.e. direct protein delivery 20, defined media 21, plasmid transfections 22 have been employed to deliver reprogramming factors to generate iPSCs. Generally, non-integrative iPSCs derivation strategies are seemingly safe for cell therapy, yet associated with challenges to produce and purify required quantities of recombinant proteins in protein-based strategies 23 , stringent steps to manipulate sensitivity of viral RNA replicase in Sendai virus delivery method 19 and are often associated with low reprogramming efficiency 24. Messenger RNA (mRNA) offers an integration-free alternative method to make footprint free iPSCs with higher reprogramming efficiency, though it is relatively laborious due to serial delivery of multiple reprogramming molecules to target somatic cells to induce pluripotency 25 ^,^ 26. In contrast, lentiviral delivery methods have the advantage over non-viral methods by transducing non-proliferative cells and better reprogramming efficiency, though cells transduced with this method are not suitable for cell therapy due to transgene integration but could be helpful for in vitro disease modeling and screening of new drugs 27. Nevertheless, the use of non-integrating viral methods and non-viral delivery methods for the derivation and long-term and large-scale culture of human iPSCs is not cost-effective. In addition to reprogramming factor and delivery methods, use of feeder cells is also crucial to maintaining the pluripotency of iPSCs. Mostly, DMD-iPSCs have been generated using mouse feeders that can be a possible cause of xenogeneic contaminations, and variability in cells and culture conditions, limiting their use in clinical applications 9 ^,^ 10 ^,^ 28. Defined culture conditions by employing human feeders obtained during different stages of development (fetal, neonatal, and adult) from various tissue sources like skin, muscle, and placenta have also been shown to support the iPSCs culture in an undifferentiated state and to bypass the problem of xenogeneic contaminations 29 ^,^ 30. Nonetheless, the inconsistency and heterogeneity are still major concerns and can be overcome by the only use of a consistent source of the human feeders. Additionally, generation of DMD-iPSCs also require supplementation of growth factors and is not cost effective for large-scale use.

Thus, no single method adequately addresses all limitations and remains constrained by the complexity, low reprogramming efficiency, heterogeneity and higher cost associated with the method used 31 ^,^ 25. In this study, we demonstrated an efficient generation of DMD-iPSCs on immortalized human feeders with improved reprogramming efficiency, and kinetics.

## Materials and Methods

**Derivation of DMD primary human fibroblast cells (hFib)**

Our study was conducted only after obtaining written informed consent from the parents or guardians of the participants and approval from the Institutional Committee for Stem Cell Research and Therapy Institutional (Ref 23/02/10-A5) and the Institute Ethics Committee All India Institute of Medical Science (Ref. IEC/NP-82/2010). Skin biopsies were taken from DMD patients ages 6-12 years old under properly administered local anesthesia were obtained from trained physician using a 6-mm punch biopsy needle. Biopsy specimens were transported to the laboratory under sterile conditions. Culture of hFib cells from skin biopsy was performed as previously described . Briefly, the dermis was separated out from the rest of the skin (epidermis, subcutaneous tissue, vascular structure) using scalpel and forceps.The dermis was cut to small pieces of explants of approximately 2mm x 2mm sizes. Few explants were then placed on 35-mm culture dish and covered with a sterile coverslip. The explants were maintained for 7-14 days in DMEM-high glucose (Gibco/Invitrogen, USA) supplemented with 10% FBS (Hyclone, USA), 2mM glutamine (Gibco/Invitrogen, USA), 1% Penicillin /streptomycin (Gibco/Invitrogen, USA). Cells from passage number 3-4 were used for reprogramming experiments. Human embryonic stem cells (KIND-1) and bFGF secreting immortalized human foreskin fibroblast (I-HFF) cells were kind gifts of Dr. Deepa Bhartiya (National Institute for Research in Reproductive Health, India) and Professor Anis Feki (Geneva University Hospital, Switzerland), respectively. These cells were cultured and maintained as described earlier 32 ^,^ 30. Primary mouse embryo fibroblast (PMEF) was commercially available and purchased from the company (Millipore, USA).

**Preparation of Feeders and mitotic inactivation**

Mouse and human feeders were plated onto 0.1% gelatin coated 6-well culture plates at a density of 4.75 x 10^5^ cells. When cells reached 80% confluency, mitomycin C (10ug/ml) (Sigma, USA) was added to the medium and incubated for 3h at 37ºC and 5%CO_2_. After incubation, the medium was aspirated, and cells were washed with buffered saline to remove any traces of mitomycin C and used immediately or cryopreserved for future use.

**Collection of Conditioned media**

I-HFF feeder cells were plated on 25cm^2^ gelatin-coated tissue culture flask at a density of 1.25x10^6^ in I-HFF expansion media. After 24h, I-HFF was mitotically inactivated by mitomycin C as described above and supplemented with basal iPSC media, composed of Knockout-DMEM (KO-DMEM) supplemented with 20% Knockout Serum Replacement (KO-SR), 1% nonessential amino acids, 2mM/ml L-glutamine, 1% penicillin and streptomycin (all media components from Gibco/Invitrogen, USA), 0.01mM β-mercaptoethanol (Sigma, USA). After 24h, conditioned medium (CM) was collected and stored after filtration at -20ºC until used for reprogramming and expansion medium of iPSCs.

**Generation of DMD-iPSCs on I-HFF and mouse feeder**

DMD-iPSCs were generated using human STEMCCA constitutive polycistronic (OKSM) lentivirus reprogramming kit (Millipore, USA) as per manufacturer’s instructions. Briefly, hFib cells were infected with the multiplicity of infection (MOI) of 1 in the presence of 6 μg/ml of polybrene (Sigma, USA). After 24h of infection, the medium was changed to fresh medium. The infected cells were maintained in hFib expansion media until day 6. After six days of infection, cells were cultured on the mitotically inactivated mouse as well as on human I-HFF feeders using standard hESC culture conditions. Cells grown on human feeders were also fed with conditioned medium from I-HFF and fresh iPSC basal medium in 1:1 ratio with 15ng/ml basic fibroblast growth factor (bFGF) (Peprotech, USA), as shown in figure (Figure1). DMD-iPSCs grown on human feeders were maintained in conditioned medium from I-HFF (I-HFF-CM).

Efficiency of iPSCs generation was calculated by the following formula:

Total number of iPSCs colonies obtained/0.1x10^5^ fibroblasts exposed to the virus x 100.

**Schematic representation of experimental approach for generating DMD-iPSCs.**
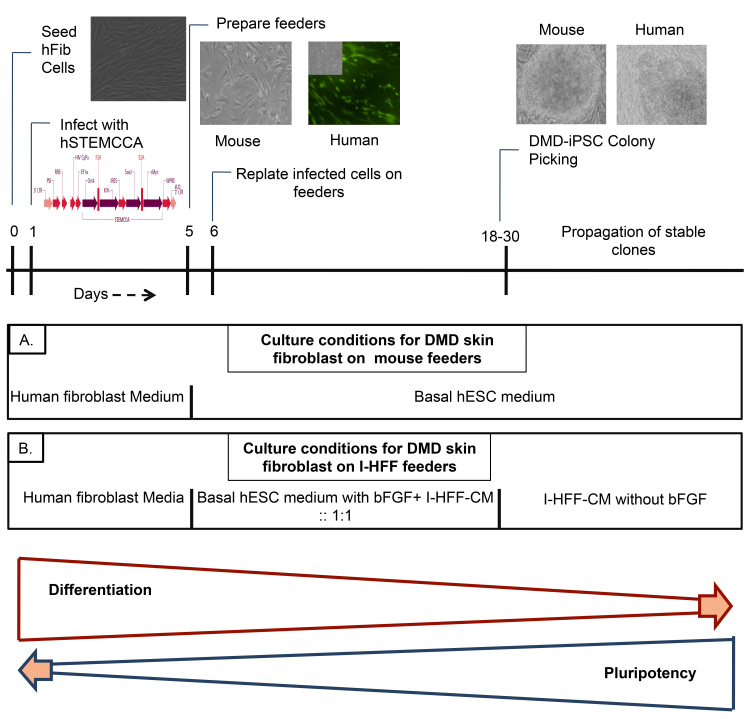
Freshly isolated DMD derived hFib cells in fibroblast expansion medium were infected with lentivirus, and 6 days post infection, maintained under hESC culture conditions till colony picking; (A) medium cocktail used during reprogramming of DMD human Fib Cells with hSTEMCCA on mouse Feeders; (B) medium cocktail used during reprogramming of DMD human Fib Cells with hSTEMCCA on immortalized I-HFF feeders.

**Passaging of DMD-iPSCs clones**

Fully reprogrammed DMD-iPSC colonies were manually picked with the help of heat-pulled glass Pasteur pipettes (Sigma, USA) under stereomicroscope (Nikon, Japan) and disintegrated into small clumps mechanically without enzymatic digestion and transferred to 24-well plates on mitomycin C treated feeders. This stage was counted as passage 1.

**Characterization of genetic defects in iPSCs cells**

DMD-hFib and DMD-iPSCs were checked for the exons deletion in dystrophin gene using Chamberlain or Beggs multiplex primer sets and PCR 33 ^,^ 34.

**Alkaline Phosphatase and Immunofluorescence staining**

Alkaline phosphatase staining was performed using alkaline phosphatase detection kit as per manufacturer’s protocol (Millipore, USA). Live staining was performed as described previously 35. Briefly, TRA 1-60 antibody (Chemicon, USA) was diluted in KO-DMEM (1X) medium at 1:100 dilutions and added into reprogramming cell culture plates and incubated for 1 hour at 37ºC. Followed by washing of unbound primary antibody using KO-DMEM (1X) media. The reprogramming cell culture plates were again incubated with appropriate secondary antibody in 1:100 dilutions for 1 hour at 37ºC. After washing, the medium was changed to routine expansion medium. The colonies were identified under an inverted fluorescent microscope and picked up for further expansion and characterization. TRA-1-60 positive colonies were considered as fully reprogrammed clones and calculated as a fraction of positive TRA 1-60 colonies to total colonies obtained.

For intracellular staining, cells were fixed with 4% paraformaldehyde and permeabilized with 0.1% Triton X-100 followed by blocking in 2% BSA for 1 h. Cells were then probed for Oct4, Sox2, Cardiac Troponin I, α SMA, Tuj1, Nestin, AFP and GATA 4 primary antibodies (Abcam, USA). Afterward, cells were further incubated with Alexa Fluor 488- or 568 tagged secondary antibodies (Molecular Probes, USA) for 1 h. Images were captured on Nikon TE 80i (Nikon, Japan) fluorescent microscope and analyzed using NIS element software.

**Gene expression analysis**

Total RNA was isolated using Trizol (MRC Inc., USA) reagent and was reverse transcribed using cDNA synthesis kit (Applied Biosystems, USA), according to the kit’s instructions. Expression of various pluripotency and differentiation markers was analyzed using reverse transcriptase-polymerase chain reaction (RT-PCR). Quantitative analysis was carried out in Realplex4 real-time PCR system (Eppendorf, USA) using SYBR green chemistry. The reaction was performed in triplicates, and internal endogenous GAPDH gene expression was used for normalization. Relative quantification was calculated using the comparative Ct method/2-ΔΔCt method. The data are presented as mean fold change.

**Analysis of pluripotency in vitro/Embryoid body (EB) formation**

For embryoid body formation, undifferentiated DMD-iPSC colonies were manually picked up and dissociated into small clumps. These clumps were allowed to grow in suspension culture using non-adherent dishes in the hESC medium with 15% FBS without bFGF. The medium was changed every other day. After 14 days, cystic EBs was further cultured on gelatin-coated dishes for spontaneous differentiation into various cell lineages using same media. At day-14 and 21 EBs were harvested for gene and protein expression studies.

**Statistical analysis**

The data were analyzed and plotted using GraphPad Prism (GraphPad Software, Inc., USA) and Windows Excel (Microsoft, Redmond, USA). Statistical significance was determined using an unpaired Student t test. The data were presented as means±SD. The p-value < 0.05 was considered significant and > 0.05 was non-significant (ns). Single asterisk and double asterisk depict different p-values p<0.05 and p<0.01, respectively.

## Results

**Human feeders improve kinetics and reprogramming efficiency of DMD-iPSCs**

Reprogramming kinetics and efficiency to generate DMD-iPSCs were checked on both human I-HFF and mouse feeders by comparing temporal kinetics and fraction of total colonies to the number of transduced cells, respectively. DMD-specific hfib cells displayed improved reprogramming kinetics on human I-HFF feeder when compared to mouse feeders, which is evident by showing increase in percentage of TRA-1-60 positive (95.5%) live staining (Figure 2A left panel) and by early appearance (12-15 days) of colonies (Figure 2A right panel). We also observed higher reprogramming efficiency (0.99+0.20 %) on I-HFF feeders in comparison to mouse feeders (0.25+0.07 %) (Figure 2B). Colonies developed on both feeders displayed morphology similar to well-established human ESC line; however, colonies grown on human feeders were more defined and compact (Figure 2C).

**Enhanced reprogramming efficiency of hFib on immortalized human feeders.**
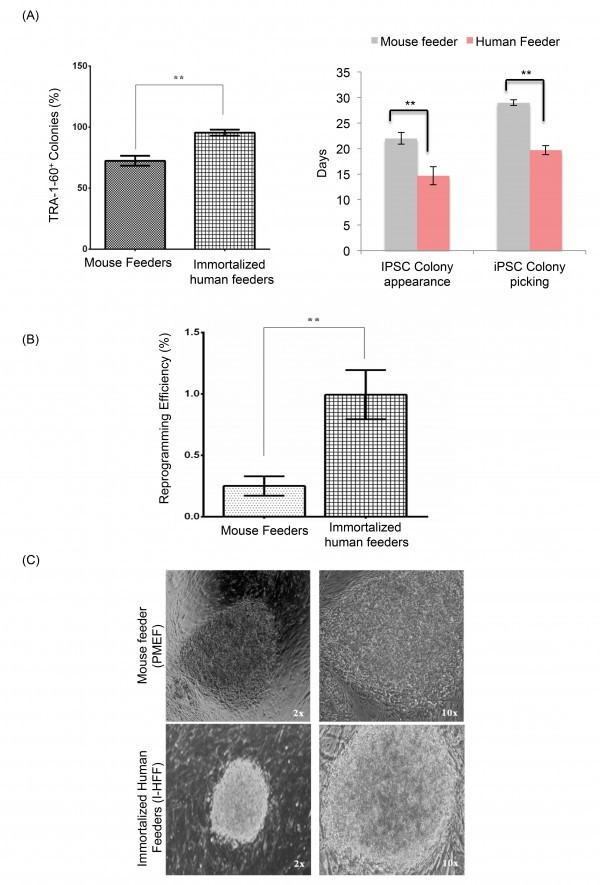
(A) Percentage of TRA 1-60 positive DMD-iPSC colonies among total colonies generated by hSTEMCCA (left panel) and temporal kinetics of reprogramming (right panel) on both mouse and human feeders. The graph represents mean of three independent experiments (n=3). Error bars show mean ± S.D, **: p<0.01; (B) Percent reprogramming efficiency of DMD-iPSCs obtained on mouse and human feeders. Data are represented as mean ± S.D, **: p<0.01, (n=3); (C) Representative pictures of DMD-iPSCs colonies derived using skin fibroblast from DMD patient on both mouse (upper panel) and human feeders (lower panel) at 2x and 10x magnifications. Resembling typical hESC morphology with well-defined borders and high nuclear to cytoplasmic ratio.

**Human feeders provide stable growth and pluripotency to DMD-iPSCs without bFGF supplementation**

Stable DMD-iPSC clones (D-iPSC 1-3) obtained on I-HFF feeders were propagated in I-HFF-CM without supplementation of bFGF and checked for the pluripotency and human embryonic stem cells (hESC) like characteristics. These clones displayed expression of pluripotency markers such as TRA-1-60, OCT4, Sox2 using immunocytochemistry and alkaline phosphatase (AP) staining (Figure 3A; 3B and 3C). Pluripotency was also confirmed by gene expression analysis using RT-PCR and quantitative real-time PCR for pluripotency markers (Figure 3D and 3E, Table 1). These clones expressed endogenous OCT4, SOX2, KLF4, c-MYC and NANOG similar to hESCs.

**Table 1:** Primer Sequences for both RT and qPCR

Primer Sequence (for both RT and qPCR)	Annealing Temp.	Product Size
Pluripotency Markers
OCT4	F 5’AGCGAACCAGTATCGAGAAC 3' ; R 5’TTACAGAACCACACTCGGAC 3’	55ºC	142bp
SOX2	F 5’AGCTACAGCATGATGCAGGA 3’ ; R5’GGTCATGGAGTTGTACTGCA 3'	55 ºC	126bp
KLF4	F 5’TCTCAAGGCACACCTGCGAA 3’; R5’TAGTGCCTGGTCAGTTCATC 3	57 ºC	105bp
cMYC	F 5’ACTCTGAGGAGGAACAAGAA 3’ ; R5’TGGAGACGTGGCACCTCTT 3’	55 ºC	159bp
NANOG	F 5’TGAACCTCAGCTACAAACAG 3’; R5’TGGTGGTAGGAAGAGTAAAG 3’	53 ºC	154bp
Differentiation Markers
i) Ectodermal
Nestin	F 5’GCCCTGACCACTCCAGTTTA 3’; R5’GGAGTCCTGGATTTCCTTCC 3’	55 ºC	200bp
βIII Tubulin	F 5’GGGATCCACTCCACGAAGTA 3’; R5’CGAGACCTACTGCATCGACA 3’	61ºC	447bp
ii) Mesodermal
RunX2	F 5’ AGAGGTACCAGATGGGACTGTGGTT 3’; R5'GGTAGCTACTTGGGGAGGATTTGTG 3’	55 ºC	199bp
Cardiac Actin	F 5’CTTCCGCTGTCCTGAGACAC 3’; R 5’CCAGACTGGAAGGTAGATGG 3’	61ºC	400bp
iii) Endodermal
AFP	F 5’TGCCAACTCAGTGAGGACAA 3’ ; R 5’TCCAACAGGCCTGAGAAATC 3’	60ºC	345bp
GATA4	F 5’TCCAAACCAGAAAACGGAAG 3’; R 5’CTGTGCCCGTAGTGAGATGA 3’	61ºC	195bp
Housekeeping
GAPDH	F 5’GAGTCAACGGATTTGGTCGT 3'; R 5’GACAAGCTTCCCGTTCTCAG 3'	57ºC	180bp

**Characterization of DMD-iPSCs without supplementation of bFGF.**
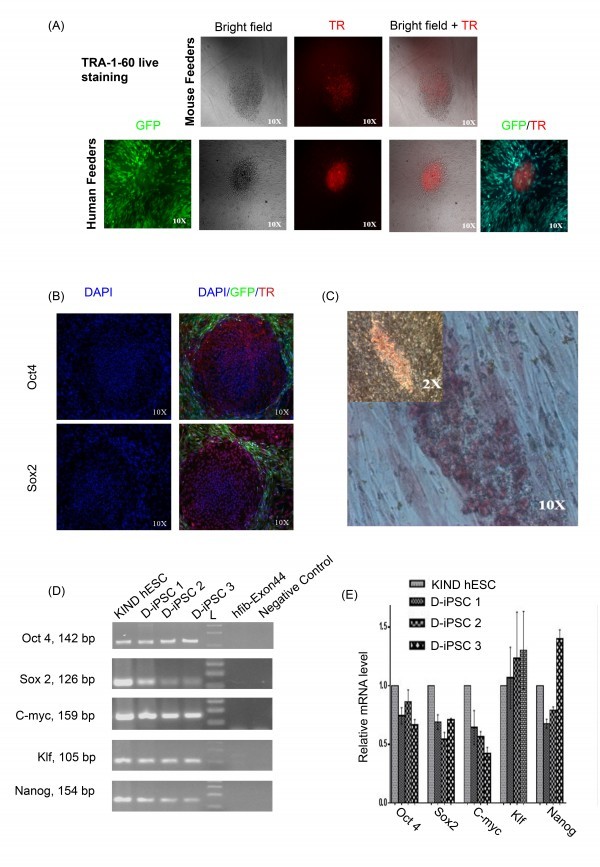
(A) “Live” Tra-1-60staining (Red) and merged with bright-field appearance of indicated DMD-iPSC clones on mouse (upper panel) and human (lower panel) feeders. Red color indicates Texas Red labeled Tra-1-60 positive cells, I-HFF are tagged with green fluorescent protein (GFP); (B) Fluorescent microscopic images showing expression of hESC undifferentiated cell markers (Oct4, Sox2- Texas Red stained positive cells) nuclei- blue DAPI stained and I-HFF is green (GFP) stained, magnification 10X; (C) Representative image of DMD-iPSC showing alkaline phosphatase staining at 2x and 10x magnifications; (D) RT-PCR analysis and (E) qRT-PCR of endogenous gene expression of pluripotent markers in DMD-iPSC clones. Primers used for Oct3/4, Sox2, Klf4, and c-Myc specifically detect the transcripts from the endogenous genes, but not from the lentiviral transgenes. The negative control is without cDNA. D-iPSC-1, D-iPSC2 and D-iPSC3 represent three different clones from DMD-iPSCs. Data is presented relative to hESCs. Expression was normalized to GAPDH. Error bars represent standard deviation of replicates (n=5).

**DMD-iPSCs show deletion in dystrophin gene**

To further confirm the DMD disease state, we did multiplex PCR for exon-44 deletion. We observed deletion of exon-44 in both non-reprogrammed hFib cells and reprogrammed DMD-iPSCs (Figure 4).

**DMD-iPSC has a deletion over exon 44 (multiplex PCR for the dystrophin gene).**
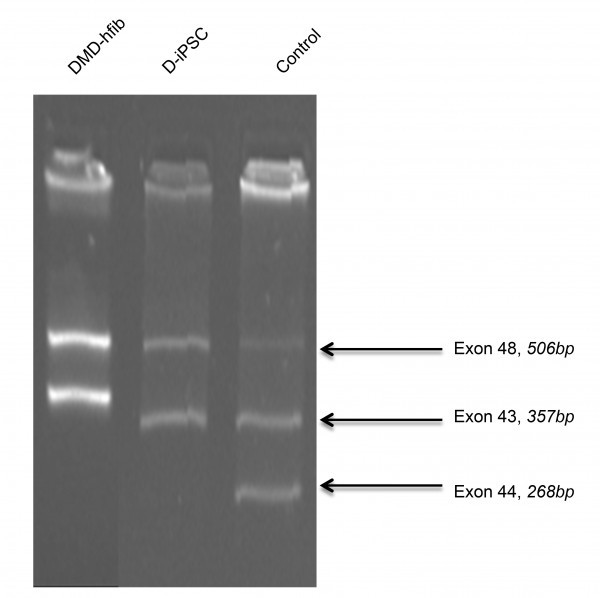
DMD-hfib is fibroblasts from patients diagnosed with DMD. The control is genomic DNA from a healthy individual. DMD-iPSCs (D-iPSCs) show deletion of exon 44 (268bp) similar to fibroblast derived from DMD patient.

**Confirmation of DMD-iPSCs pluripotency by embryoid body formation**

We confirmed the ability of all DMD-iPSCs clones to differentiate into three germ layers in vitro using the embryoid body (EB) assay. All iPSC clones displayed expression of all three lineages ectoderm, mesoderm, and endoderm markers, as evident by immunofluorescence and RT-PCR (Figure 5A; 5B and 5C). Embryoid bodies derived from DMD-iPSC clones displayed absence of pluripotency markers as quantified by qRT-PCR (Figure 5D).

**In-vitro differentiation of D-iPSC without supplementation of exogenous bFGF.**
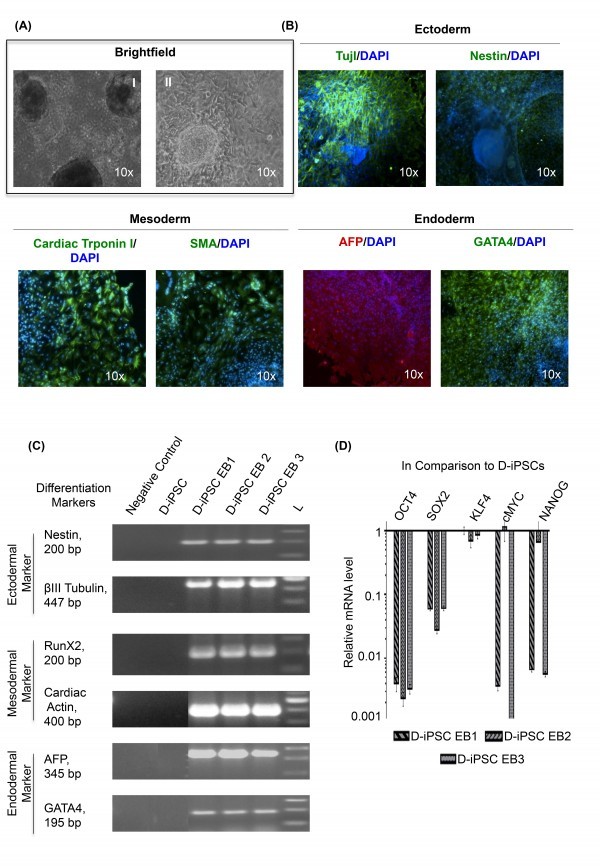
(A) Representative images of cystic embryoid body (EB) in suspension culture (I) and their spontaneous differentiation (II) into three germ layers (magnification 10x); (B) Immunostaining of 21 days old EBs derived from D-iPSC revealed expression of ectodermal (Tuj 1 and Nestin), mesodermal (Cardiac troponin 1 and SMA), and endodermal (AFP and GATA4) marker proteins. Tuj 1, Nestin, Cardiac troponin 1, SMA and GATA4 are conjugated with fluorescein isothiocyanate (FITC); AFP protein is conjugated with Texas red. (Magnification 10x) (C) RT-PCR analysis of various differentiation markers for the three germ layers by EB mediated differentiation; D-iPSC is negative for expression. (D) Quantitative analysis of endogenous gene expression of pluripotent markers in D-iPSC EBs using qRT-PCR. Data is presented relative to D-iPSC clones. The expression is normalized to GAPDH. Error bars represent standard deviation of replicates (n=5). D-iPSC EB1, D-iPSC EB2, and D-iPSC EB3 represent embryoid bodies obtained from three different DMD-iPSCs clones.

## Discussion

The field of iPSC is very promising when it comes to basic, pre-clinical and clinical aspects of regenerative medicine. However, the generation of DMD disease-specific iPSCs (DMD-iPSCs) is surrounded by various impeding factors like inefficiency, slow kinetics, safety issues, and the high cost of culture maintenance 9 ^,^ 10 . Here we reprogrammed DMD skin fibroblast in less than three weeks, from infection of somatic cells to iPSCs colony expansion with enhanced reprogramming efficiency and kinetics. Thus, enables the generation of DMD disease-specific iPSCs in reliable, cost-effective, and timesaving manner. To examine the effect of reprogramming approach and delivery methods we checked reprogramming efficiency and appearance of colonies after transduction of DMD fibroblast using integrated viral monocistronic and polycistronic methods and integration-free monocistronic viral methods. Use of polycistronic lentiviral vector improved the appearance of reprogrammed colonies and reprogramming efficiency of DMD patient-derived fibroblasts.

**Figure:**
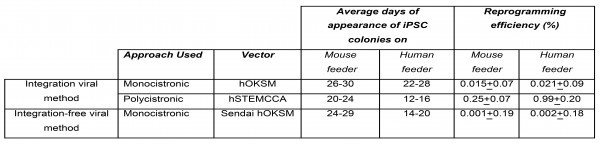
**Supplementary Table 1: **Reprogramming efficiencies and kinetics of DMD derived fibroblast on both human and mouse feeders obtained by employing monocistronic and polycistronic integrating Lentiviral method and monocistronic integration-free Sendai virus approach. Data shown represent the mean ± SD of three independent experiments (n=3).

Experimentally, we were able to derive iPSCs clones with the multiplicity of infection (MOI) as low as one (Supplementary Table 2) as previously demonstrated by others 16.

**Figure:**
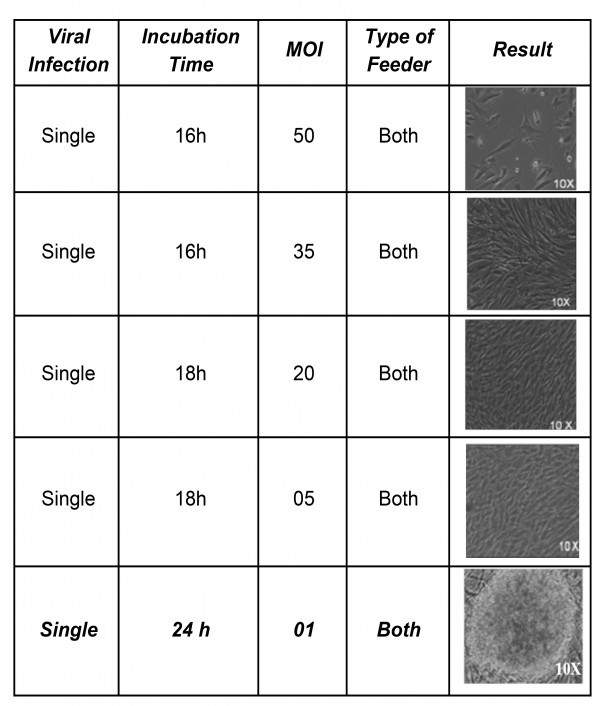
****Supplementary Table 2: ****Depicting the variations at a glance adopted for standardization for reprogramming of hFib cells for the efficient generation of DMD-iPSCs using polycistronic approach at the different multiplicity of infections (MOIs). Representative picture of iPSC colony obtained after infection of DMD patient-derived fibroblast with hSTEMCCA viral particles at MOI of 1.

We used bFGF secreting immortalized human feeder in our study that has been shown already to secrete stable amounts of bFGF to support growth and maintenance of induced pluripotent stem cells, though not has been used to generate DMD-iPSCs 30. Supplementation of bFGF has been shown dispensable for the self-renewal and survival of hESCs by direct activation of signaling pathways, indirectly stimulating autocrine effect and paracrine network 36 ^,^ 37. Many reports have demonstrated the cooperation of both TGF-β and IGF-II with the bFGF pathway in maintenance of hESC pluripotency by establishing a regulatory stem cell niche 38 ^,^ 39. In another study of ours (unpublished data), we have also detected the significant amount of both (TGF-β and IGF-II) the molecules in conditioned medium of I-HFF cells as compared to other primary human and mouse fibroblast feeders (data not shown). To further substantiate the possible role of secreted molecules we compared reprogramming efficiency and kinetics of DMD fibroblast on immortalized human feeders in basal hESC medium with or without I-HFF-CM. The addition of conditioned medium significantly improved the kinetics and reprogramming efficiency of DMD fibroblasts (Supplementary Table 3).

**Figure:**
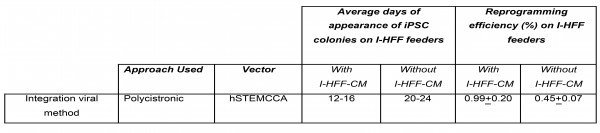
****Supplementary table 3: ****Reprogramming efficiencies and kinetics of DMD derived fibroblast obtained on I-HFF feeders with or without the addition of conditioned medium (I-HFF-CM) by employing polycistronic lentiviral approach. Data shown represent the mean ± SD of three independent experiments (n=3).

Several types of feeder cells MEF 40, human placenta 41, human fetal muscle and adult fallopian tube fibroblasts 42 ^,^ 43 , and human dermal fibroblast 44 have been used for reprogramming and maintenance of iPSCs. However, these feeders cannot be readily isolated, support only for few passages, and also bear a risk of infection, and contamination 44 ^,^ 45 ^,^ 46. Moreover, hESC when grown in the presence of xenogeneic feeders, are contaminated with Neu5Gc, a non-human sialic acid that can induce an immune response in humans and limit their use in therapeutic applications 45. Therefore, we included immortalized human feeder to overcome these limitations of heterogeneity and inconsistency.

To the best of our knowledge, we for the first time demonstrated that bFGF secreting human I-HFF feeders together with conditioned medium (I-HFF-CM) promote the reprogramming efficiency and kinetics of DMD-iPSCs, appeared early in 17-20 days and developed into fully reprogrammed colonies earlier than colonies grown on mouse feeders, as confirmed by percent of TRA-1-60 positive DMD-iPSC colonies (Figure 2A), and calculated percent reprogramming efficiency (Figure 2B). Generated DMD-iPSCs developed on human feeders together with I-HFF-CM showed similar morphology to human ES cells (Figure 2C).

Conditioned medium (I-HFF-CM) alone was sufficient to maintain DMD-iPSCs in undifferentiated states without exogenous bFGF supplementation (Figure 3A and 3B). We confirmed the pluripotency of these cells using alkaline phosphatase (AP) staining (Figure 3C), pluripotency markers by RT-PCR (Figure 3D) and qRT-PCR (Figure 3E). DMD-iPSCs generated on human feeders was also confirmed the DMD-specific genotype of their parental fibroblast cells for exon deletion using multiplex PCR (Figure 4) as reported earlier 9. Similar to discussed clones from one patient, we also successfully generated and characterized DMD-iPSCs from different patients to rule out intra-patient variability (Supplementary Table 4).

**Figure:**
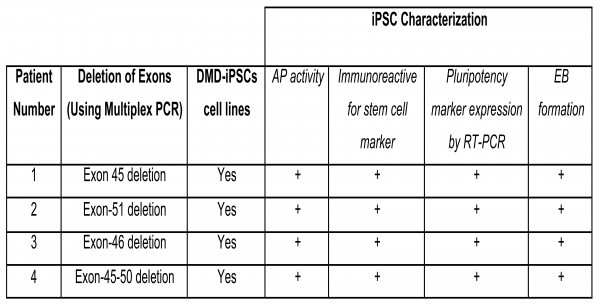
**Supplementary table 4: **Characterization of established human DMD-iPSCs cell lines from various DMD donors.

These DMD specific iPSCs was also differentiated in to embryoid body that was confirmed by immunostaining (Figure 5A), RT-PCR (Figure 5B) and qRT-PCR (Figure 5C). Hence, the use of I-HFF-CM provided an insight in the role of supportive factors released by feeders in the successful and complete reprogramming.

These DMD-specific iPSCs was also differentiated into the embryoid body formation that was confirmed by immunostaining (Figure 5A), RT-PCR (Figure 5B) and qRT-PCR (Figure 5C). Hence, the use of I-HFF-CM provided an insight into the role of supportive factors released by feeders in the successful and complete reprogramming.

On the basis of above observations, it is confirmed that addition of I-HFF-CM could enhance the reprogramming and kinetics of DMD fibroblast, and pluripotent characteristics of DMD-iPSCs can be preserved using I-HFF-CM without supplementation of additional bFGF, making stem cell culture more economical, and less time-consuming, thereby reducing the time for feeder cell preparation. This study laid the preliminary findings that can be further extended to combine the ex-vivo gene and autologous cell therapy to exploit the therapeutic way to treat DMD that avoid immune rejection with sufficient engraftment ability 40 ^,^ 4. Also, the immaculate expertise in patient-derived iPSCs can accelerate the possibility of personalized medicine in the clinical arena.

## Competing Interests

The authors indicate no potential conflicts of interest.

## Author Information

**Corresponding Author: **Sujata Mohanty, Ph.D.****

**Complete Mailing Address: **Stem Cell Facility, 1^st^ Floor ORBO Complex,

All India Institute of Medical Sciences,

Ansari Nagar, New Delhi-110 029

**Telephone and Fax Numbers: **+91-11-2659 3085 (Telephone)

+91-11-2658 8663 (Fax)

**Email Id: **drmohantysujata@gmail.com****

**Pooja Teotia**: teotiapooja@gmail.com

**Sujata Mohanty**: drmohantysujata@gmail.com

**Madhulika Kabra**: madhulikakabra@hotmail.com

**Sheffali Gulati**: sheffaligulati@gmail.com

**Balram Airan**: iactscon_2004@yahoo.co.in

**Short Title:** *i-PSCs for human* *Duche* *nne muscular dystrophy.* ****
